# Improved thermal conductivity of polyurethane (PU)-/SiC composite fabricated via solution casting method and its mechanical model for prediction and comparison

**DOI:** 10.1016/j.heliyon.2023.e15571

**Published:** 2023-04-18

**Authors:** Patcharapon Somdee, Manauwar Ali Ansari, Tamas Szabo, Kalman Marossy

**Affiliations:** a Institute of Ceramic and Polymer Engineering, University of Miskolc, Miskolc-Egyetemvaros, 3515, Hungary; b BorsodChem Zrt., Kazincbarcika, 3700, Hungary; c Department of Materials Engineering, Rajamangala University of Technology Isan, 744 Suranarai Road, Muang-Nakhon Ratchasima, 34000, Thailand

**Keywords:** Polyurethane, Polymer matrix composites, Mechanical modelling, Silicon carbide, Thermal conductivity, Differential scanning calorimetry

## Abstract

Polymer composites having high thermal conductivity (TC) gained great interest, including the advancement of electronic devices to become more functionalized, scaled, and integrated. In view of these, herein, highly thermal conductive polyurethane (PU)-/SiC composites are fabricated via the solution casting method. Silicon carbide is used as the filler in both flexible and rigid-polyurethane matrices to enhance the value of TC for electronic applications. A novel model has also been developed based on the Coran-Patel model for analysis and comparison of TC of as-synthesized composites. Calculated thermal conductivities by the model are found to be consistent with the experimental results. The highest measured TC for flexible as well as rigid-PU composites is 0.521 and 0.542 Wm^−1^K^−1^ representing improvements of 106% and 87% over their pure equivalents, respectively. SEM and DSC techniques are employed to analyze the samples' morphology, and other thermal properties, respectively.

## Graphical abstract

**Figure:**
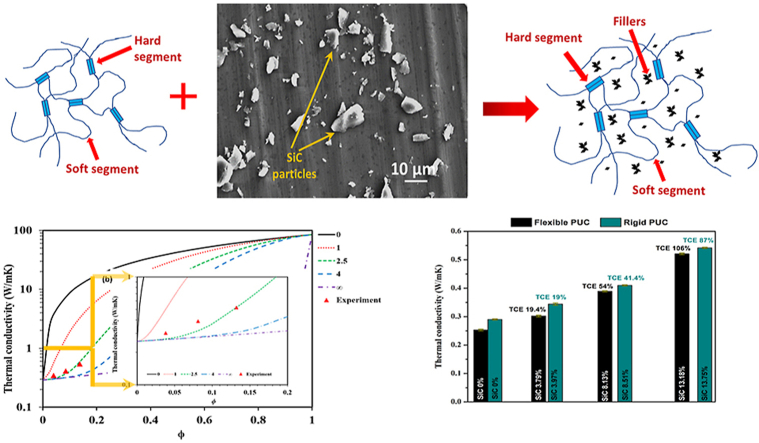

## 1 Introduction

High thermal conductivity polymers are required for several applications such as microelectronics, heat exchange, energy utilization, and so on [1,2]. Therefore, polymer composite is the one method for increasing thermal conductivity value. For this purpose, many high TC fillers such as ceramic particles [[3], [4], [5], [6], [7], [8], [9], [10], [11], [12], [13], [14], [15], [16], [17], [18], [19]], metals [[20], [21], [22], [23], [24]], nanophase material [9,[25], [26], [27]], etc. can be included into the polymer-matrices.

It has already been found that the micro-phased silicon carbide particle is a striking 3D filler for high power and high temperature uses mainly in electronic industries. This is because of its extraordinary features such as a room temperature-huge thermal conductivity of ∼390 Wm^−1^K^−1^ and a lower coefficient of thermal expansion of ∼4.0 ppm/K (value closely resembles Si chip of ∼3.5 ppm/K in the temperature range 300–673 K), etc. [28].

Polyurethane is a special polymer that can be produced and applied to a variety of applications such as foam, rigid polyurethane, elastomer, thermosetting glue, and so on [29]. The specialty of polyurethane is the hard segments (HS) as well as soft segments (SS) formed in its structure. The HS are formed by short diols (chain extender) and isocyanates while the SS are comprised of long polyols. Polyurethanes' properties can easily be improved by changing the molecular chain structure of the SSs and HSs [30]. The chain extender (MEG) is a factor that plays a crucial part in the synthesis of PUs. It has weak physical characteristics and often does not display microphase separation when generated by directly reacting polyol and diisocyanate. without the chain extender. Good mechanical characteristics of polyurethane may derive from a chain extender that increases the length of hard segments and hard segment segregation. Further, by controlling the chain extender amount and hence tunning of HS and SS could produce either flexible or rigid polyurethane elastomers (PUEs) [31].

Hitherto, a number of PU composites made up of organic or inorganic with micro or nano-phase fillers have been reported recently and showed an increase in TC over their pure equivalents, respectively. These were based on fillers for example, boron nitride (BN) [[32], [33], [34], [35]], alumina sphere-coated graphene [36], silane-modified Al_2_O_3_ [37], magnetite (Fe_3_O_4_) [38], hemp fiber (HF) [39] cellulose nanocrystals (CNC) [25], 2D d-Ti_3_C_2_ [40], SiO_2_ coated- MWCNT, *p*-MWCNT, and hyperbranched poly-urea-urethane grafted multiwalled carbon nanotubes (HPU-MWCNTs) [26,41], etc.

Moreover, several theoretical models for predicting polymer composite thermal conductivity were studied since the 19th century, for example, Cheng-Vachon, and Nielsen-Lewis models [42,43], etc. Their theoretical predictions of thermal conductivity have been revealed so far consistent with the experimental findings [44] but there are some limitations of the theoretical models when the filler loading is higher. However, TC enhancement of both (flexible as well as rigid-PUEs) by using SiC-microparticles and their theoretical predictions have not been studied yet.

In this work, for the first time flexible, and rigid-polyurethane/-SiC composites with enhanced TC, were prepared using varying chain extender loading via simple solution casting method. A novel thermal conductivity model has been also derived based on the Coran-Patel model for the comparison and prediction of TC of as-synthesized composites. To examine the inner microstructure, and morphologies of the composites, scanning electron microscopy has been used. DSC study outcomes for all samples were also provided and discussed.

## 2 Materials and method

### 2.1 Materials

The main polyether polyol in this study is Caradol MC28-02. It has 6000 g-mol^−1^ nominal molecular weight, and its viscosity and density are 1130 cP and 1021 gcm^−3^, respectively. diphenylmethane-4,4′-diisocyanate (MDI) of type ONGRONAT XP 1117 having the average molecular weight and an equivalent weight of about 266 g-mol^−1^ and 127.33 g-mol^−1^, respectively used as isocyanate. Its viscosity shows around 5–25 cP at 25 °C, and the NCO value is around 31.5–33.5 wt%. Monoethylene glycol having a molecular weight of about 62.07 g-mol^−1^ is used as the chain extender. Besides, a polyol of type ALCUPOL D0411 with lower molecular weight (400 g-mol^−1^), hydroxyl points (270 mgKOH-g^−1^), and viscosity (86 cP at RT) is employed in the synthesis. Dabco 33-LV(Db) was used as a catalyst. Its viscosity and specific gravity are 125 cP and 1.03 gcm^−3^, respectively at room temperature. The last chemical constituent is the zeolite type (Finmasorb 430 PR), used as the moisture scavenger.

For the high thermally conductive filler, silicon carbide (SiC) powder; purchased from Minerals Water, United Kingdom having the particle sizes are around 7 μm.

### 2.2 Preparation of composites

**Fig. 1:**
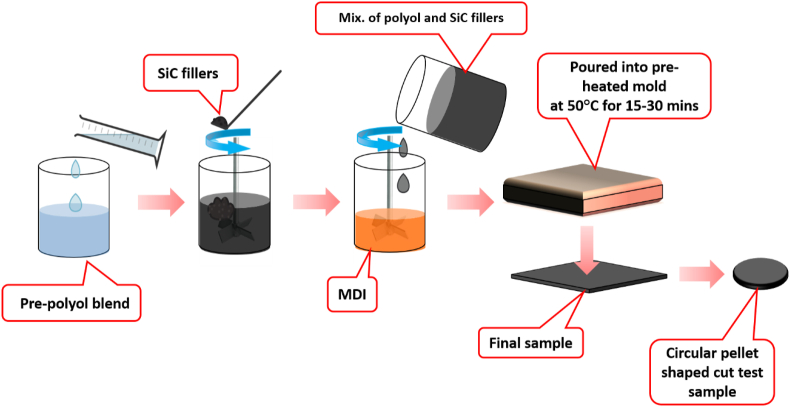
Schematic for fabrication steps of polyurethane/-SiC composites.

The proportion of the chain extender was varied for the fabrication of both flexible and rigid-polyurethane matrices. One formula was prepared for the flexible-PU at 10 phr chain extender content while the others for the rigid-PU at 30 phr chain extender content. Then, the polyurethane composites were prepared. Fig. 1 provides an overview of the fabrication process. The filler (SiC, ∼6.587 μm) was dried at 80–90 °C for up to 3 days before mixing into the pre-polyol blend. The pre-polyol component consists of polypropylene glycol, monoethylene glycol, polyether polyol, amine catalyst, and moisture scavenger as listed in Table 1. SiC-microparticles were added and mixed in the pre-polyol blend. The mixture was blended using a stirrer on about 1000 rpm for 10 min. Then, the air bubbles inside the mixture were removed by means of the vacuum pump running for around 90 min prior to mixing with the MDI. It was mixed in the pre-polyol and fillers mixture by shear-mixed over 8–15 s on about 1000 rpm, then inherently poured into an already heated mould having temperature of 50 °C. The curing time is about 15–30 min. After that, the composite sheets of 1 mm and 4 mm thickness were detached from the molds and kept at room temperature (23 °C) for up to 3 days prior to making the test specimens.

**Table 1:** The constituents of polyurethane composites formulation.

Constituents	Trade Name	Content (in phr^a^)
Polyether polyol (or PPG)	Caradol MC28-02	100
Polypropylene glycol (or PPG-400)	Alcupol D-411	5
Chain extender (Monoethylene glycol)	MEG	10, 30
Moisture scavenger (H_2_O removal)	Finma-Sorb 430 PR	5
Catalyst (1,4-Diazabicyclo [2.2.2] octane)	Dabco 33-LV(Db)	0.3–2.4
4,4′ diphenylmethane diisocyanate (MDI)	ONGRONAT XP 1117	22–50
Silicon carbide (SiC)	Size ∼ 6.587 μm	0 - 121 (0–14^b^)

a Part per hundred resins.

b By volume percent.

### 2.3 Characterization

Scanning electron microscopy (SEM, ZEISS EVO-MA10, Germany) was utilized to examine the internal microstructure and surface morphology of the composites. A coating of fine particles of gold was applied to all the samples before scanning. Thermal conductivity of samples was measured using the thermal conductivity analyzer (C-THERM, TCi, Canada). The test specimen for thermal conductivity testing should be thicker than 5 mm. C-THERM TCi uses the source of the altered transient plane approach. Differential scanning calorimetry (DSC823e, METTLER, Switzerland) was employed for the analysis of other thermal properties of the prepared samples.

### 2.4 A mathematical model for predicting composites’ thermal conductivity

**Fig. 2:**
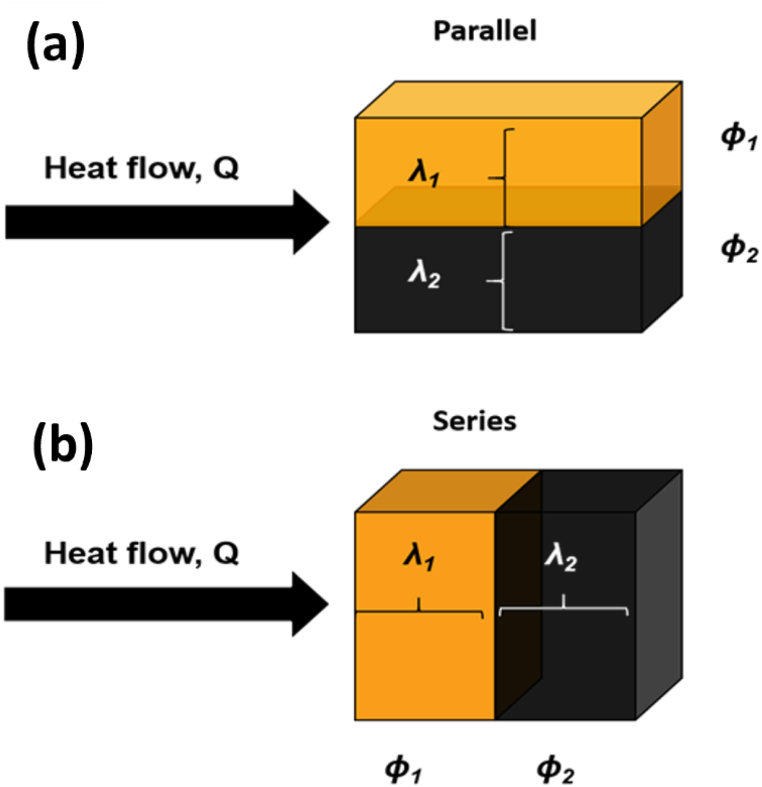
Schematic representation of heat conduction for: (a) parallel connection, and (b) series connection.

There are several kinds of thermal conductive composites accessible. The maximum and the minimum thermal conductivities are achieved when filler and polymer are either connected in parallel or series fashion in the direction of heat flow (parallel and series conduction) Fig. 2(a) and (b) [45]. The TC expression for both types of composites can be given as:

(1) $$\lambda =V_{1}\lambda_{1}+V_{2}\lambda_{2}\;parallel\;conduction$$

(2) $$\lambda ={\left[\left(\frac{V_{1}}{\lambda_{1}}\right)+\left(\frac{V_{2}}{\lambda_{2}}\right)\right]}^{-1}\;series\;conduction$$

where λ, λ_1_, and λ_2_ are the TC of; composite, component-1, and component-2, respectively. While V_1_, and V_2_ indicate volumes of component-1, and component-2, respectively.

If component-1, and component-2 represents soft (polymer) and hard (filler) phase of the composites, respectively and $\lambda_{u}$, $\lambda_{l}$ represents upper and lower TC limits, and replacing volume (V) by volume fraction (φ) then Equation (1) and (2) can also be written as:

(3) $$\lambda_{u}=\varphi_{S}\lambda_{S+}\varphi_{H}\lambda_{H}\;Parallel$$

(4) $$\lambda_{l}={\left[\left(\frac{\varphi_{S}}{\lambda_{S}}\right)+\left(\frac{\varphi_{H}}{\lambda_{H}}\right)\right]}^{-1}\;Series$$

**Fig. 3:**
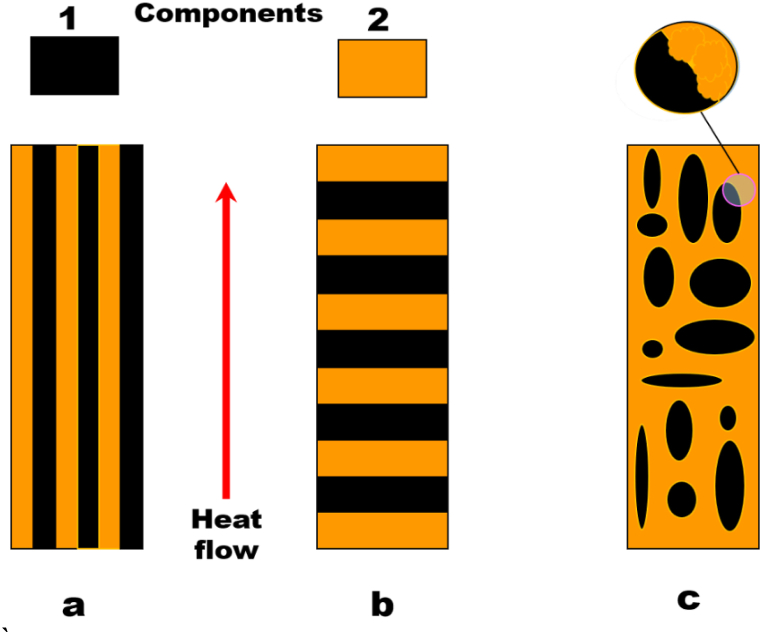
Schematic arrangement of (a) equal temperature gradient, (b) equal heat flow models, and (c) represents a real dispersed two-phases system.

The former model describes a constant temperature gradient type and the latter one a constant heat flow type. This applied model had been estimated thermal conductivity in a parallel and serial arrangement. Fig. 3(a) schematically shows the equal temperature gradient model, which leads to the TC's upper limit, while Fig. 3(b) represents an equal heat flow model, which results in the lower limit of the TC.

Equation (3) and (4) are formally identical to mechanical models of Coran-Patel [46], used for modulus (M) calculation of the same two-component type composites (parallel and series). Replacing λ by M in Equation (3) and (4) demonstrate,

(5) $$M_{u}=\varphi_{S}M_{S+}\varphi_{H}M_{H}$$

(6) $$M_{l}={\left[\left(\frac{\varphi_{S}}{M_{S}}\right)+\left(\frac{\varphi_{H}}{M_{H}}\right)\right]}^{-1}$$

where M_u_, M_l_, M_H_, and M_S_ are the upper limit of modulus, the lower limit of modulus, the modulus of the pure-hard, and -soft phases, respectively. φ_H_ and φ_S_ are the volume fractions of respective hard, and soft phases of composite.

In addition, a real dispersed two-phase-system is shown schematically in Fig. 3(c). According to Coran-Patel, the overall modulus equation using Equation (5) and (6) for this system can be given as:

(7) $$M=M_{l}+\varphi_{H}^{n}\left(n\varphi_{S}+1\right)\left(M_{u}-M_{l}\right)$$

Where M is the modulus of the composite, and n is an adjustable parameter (or an index) for modulus’ upper and lower bound. Due to the similarities, Equation (7) may also be used to evaluate thermal conductivity and is written as:

(8) $$\lambda_{C}=\lambda_{l+}\varphi_{H}^{n}\left(n\varphi_{S}+1\right)\left(\lambda_{u}-\lambda_{l}\right)$$

where λ_C_, λ_u_, and λ_l_ are the TC of the composite, the TCs’ upper, and lower limit, respectively. φ_S_ and φ_H_ are the volume fractions of soft phase (polymer) and hard phase (filler), respectively. n is the index (or characteristic exponent). If n = 0 that means the TC of composite has an upper limit (Equation (3)) and n = 100 means it has a lower limit (Equation (4)). A small computer program was written to do the computations where n = 100 considered as ∞ due to the error <0.01%. Moreover, the TC of SiC-microparticles ($\lambda_{H}$) was calculated about 85 Wm^−1^K^−1^.

## 3 Results and discussion

### 3.1 Microstructure and morphology

**Fig. 4:**
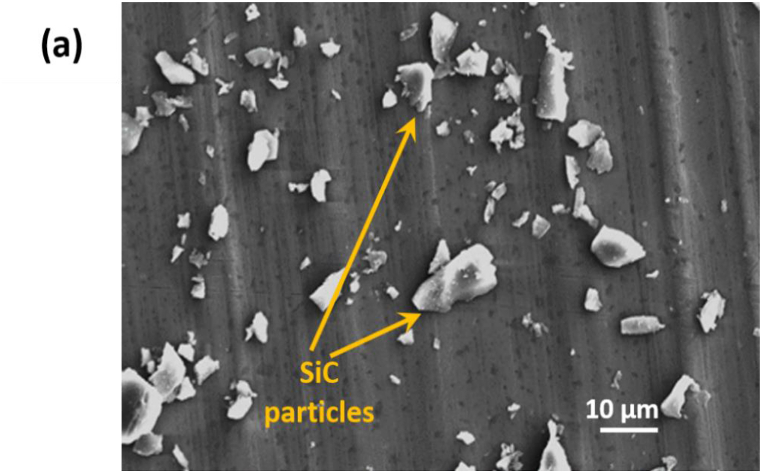

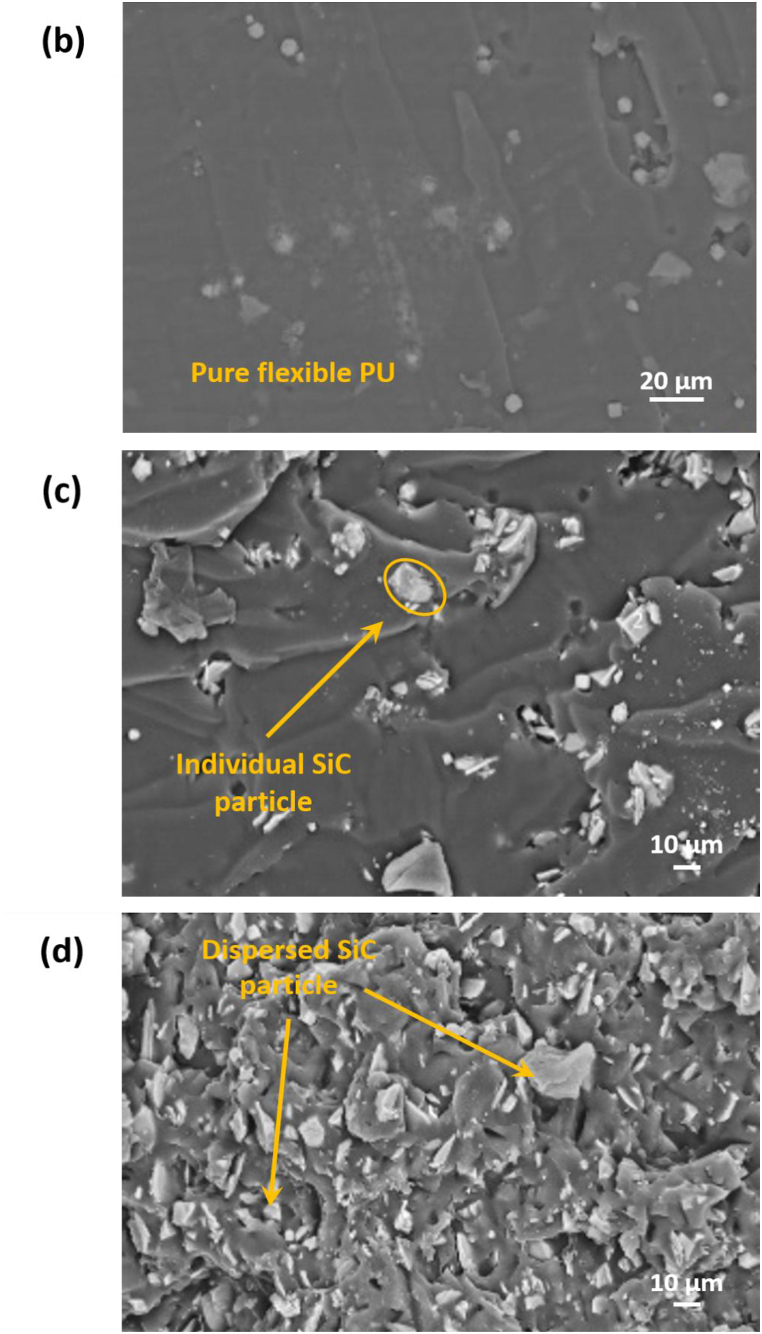

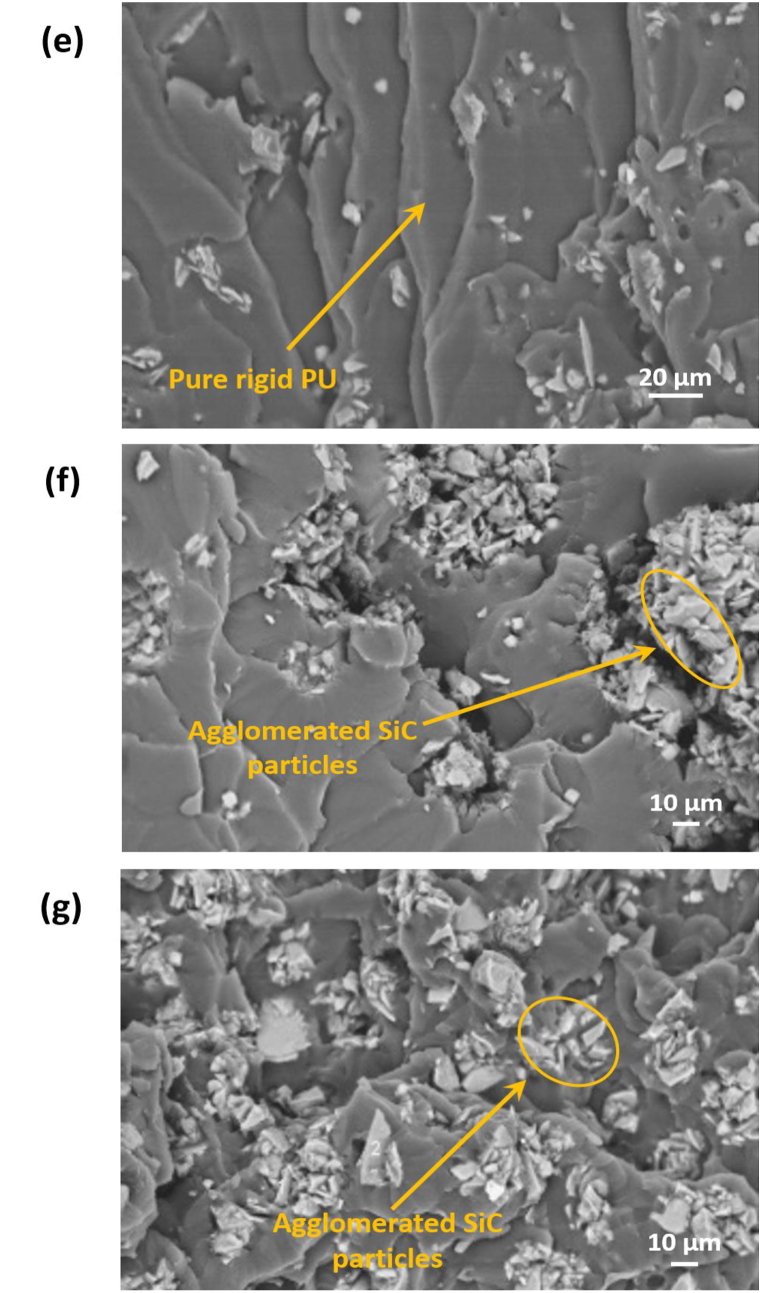
SEM images of: (a) SiC-microparticles (nominal magnification of 1.0k × ); (b) pure flexible-PU (c) flexible-PU with SiC 3.79 v/v% (d) flexible-PU with SiC 13.18 v/v%; (e) pure rigid PU (f) rigid-PU with SiC 3.97 v/v% (g) rigid-PU with SiC 13.75 v/v% (nominal magnification of 500 × ).

Fig. 4(a) represents the SEM image of SiC-microparticles utilized in this study. Fig. 4(b) and (e) show the microstructure and morphology of pure (0 v/v% SiC), flexible, and rigid-PU. SEM images of both (flexible PU, and rigid-PU) matrices with SiC fillers have evidently indicated the existence of two phases, namely the matrix and SiC- microparticles shown in Fig. 4(c), (d), 4(f), 4(g). SiC-microparticles are evenly dispersed in the flexible -PU matrix. For rigid-PU matrix, SEM image shows the agglomeration of microparticles in the matrix at 3.97 v/v% as revealed in Fig. 4(f). It also shows the effect of high viscosity in the rigid-PU matrix and the incompatibility effect between SiC and matrices which resulted in TC having the almost same value as the flexible one.

### 3.2 Thermal characterization

#### 3.2.1 Thermal conductivity

**Fig. 5:**
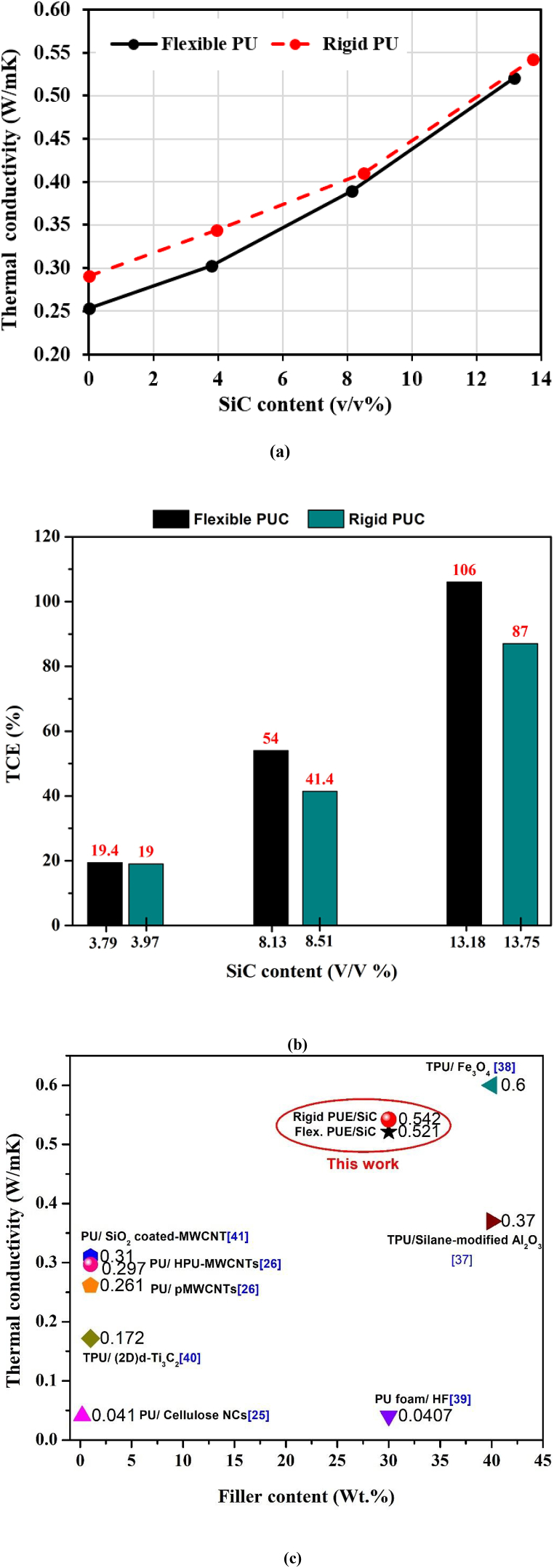
(a) TC of flexible as well as rigid-PU composite samples with various content of SiC-microparticles, and (b) TCE (thermal conductivity enhancement) of both types of PU-composites with SiC content compared to pure-PU; (c) comparison of maximum TC values of current work with various PU/fillers composites recently reported.

The TC of PU-/SiC composites was tested by varying the SiC content between 0 and 14 v/v% in both flexible, and rigid-PU matrices shown in Fig. 5(a) and (b). The TC is likely enhanced with raising SiC content in flexible as well as rigid-PU. The TC for pure flexible-PU was observed about 0.253 Wm^−1^K^−1^. With the addition of 3.79 v/v% of SiC, TC of flexible-PU composite increased to 0.302 Wm^−1^K^−1^ (19.4% enhancement). At 8.13 v/v% SiC contents, it increased to 0.389 Wm^−1^K^−1^ (54% enhancement). Further, increasing the SiC content up to 13.18 v/v% TC value reached 0.521 Wm^−1^K^−1^and revealed a significant enhancement of 106% compared to pure flexible-PU. For pure rigid-PU, the measured TC was 0.290 W/mK. By the inclusion of SiC 3.97 v/v% TC increased to 0.344 Wm^−1^K^−1^ (19% enhancement). At 8.51v/v% SiC it reaches 0.410 Wm^−1^K^−1^ (41.4% enhancement). Further increasing the SiC content up to 13.75 v/v% TC value of rigid-PU composite reached 0.542 Wm^−1^K^−1^ (87% enhancement). These results are well supported by earlier works of literature [37,38]. These results can be interpreted by the high TC value of SiC microparticles and which in turn results in the even dispersion of SiC microparticles in both the flexible as well as rigid PU matrices. Besides, improving the TC of this composite could be the high phonons propagation by SiC microparticles through a boundary [9].

Furthermore, comparing the experimental outcomes of the TC for flexible as well as rigid-PU samples with different content of SiC filler Fig. 5(a) and (b), the rigid-PU/SiC composite have higher TC at low SiC content because of its high physical crosslinking into the matrix and effect of the good dispersion of SiC-microparticles in the rigid matrix. At higher SiC content, TC of rigid PU shows almost the same value as flexible-PU matrix due to high viscosity of composite. Therefore, the composites have some agglomeration of SiC and might be created foamed structures during forming. Unfortunately, SiC content higher than 14 v/v% does not easily mix into the PU-matrix via solution mixing method. Also, the incompatibility between SiC and both the PU-matrices has a strong effect on thermal conductivity that means it is difficult to get close to the theoretical zero-line. Moreover, the obtained TC of this work can be compared to various polyurethane or fillers-based composites reported previously. Fig. 5(c) shows a comprehensive comparison.

**Fig. 6:**
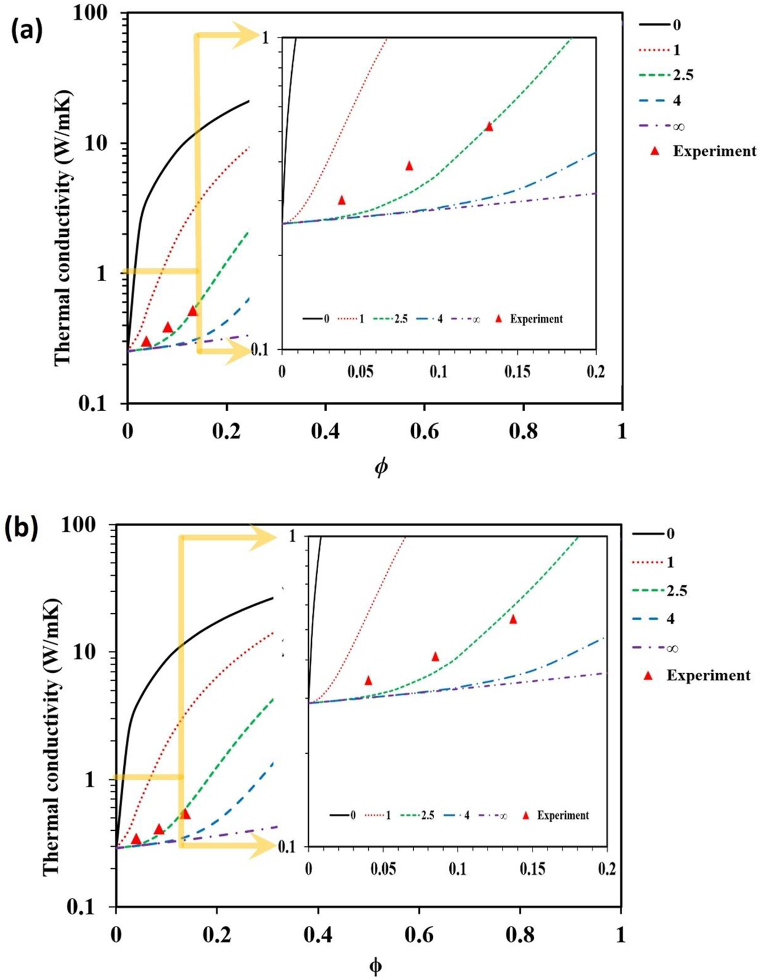
Thermal conductivity modeling curves and experimental results of: (a) flexible-PU with different SiC contents, and (b) rigid PU with different SiC contents.

The relationship between the filler loading and thermal conductivity from the experiment was compared to the theoretical model Equation (8). A total of five n values viz. 0, 1, 2.5, 4, and 100 (Table 2) were selected and plotted the corresponding TC curves shown in Fig. 6(a) and (b). It was found that the curve for n = 2.5, more closely satisfied the experimental outcomes of both types of samples. Astonishingly, the computed thermal conductivities by the model are found to be closely fitting with the experimental outcomes. It is found that this novel model can be further applied for the prediction and comparison of TC of any two-phase polymer composites.

**Table 2:** Various n input values and their equivalent output equations produced from the model Equation (8).

n	λ_C_ (Wm^−1^K^−1^)
0	λ_u_
1	$\lambda_{l}+\varphi_{H}\left(\varphi_{S}+1\right)\left(\lambda_{u}-\lambda_{l}\right)$
2.5	$\lambda_{l}+\varphi_{H}^{2.5}\left({2.5\;\varphi }_{S}+1\right)\left(\lambda_{u}-\lambda_{l}\right)$
4	$\lambda_{l}+\varphi_{H}^{4}\left({4\;\varphi }_{S}+1\right)\left(\lambda_{u}-\lambda_{l}\right)$
100	≈ λ_l_
∞	λ_l_

#### 3.2.2 Differential scanning calorimetry (DSC)

**Fig. 7:**
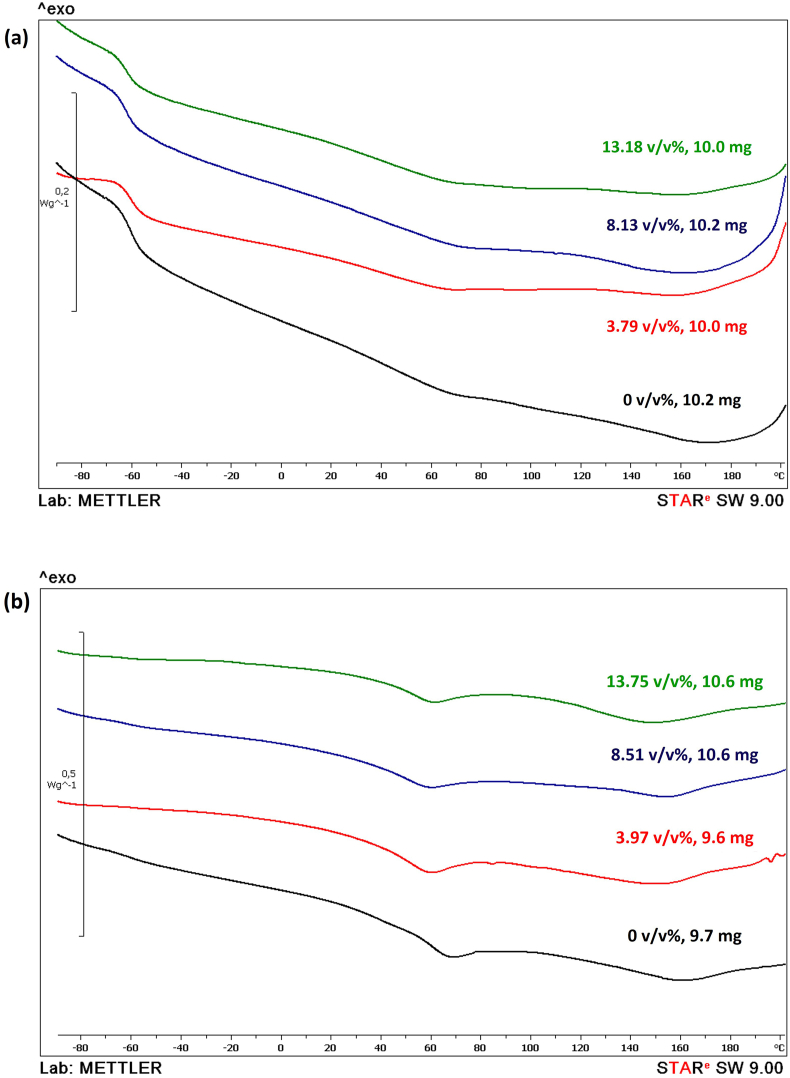
DSC curves of: (a) flexible-PU with different SiC contents, and (b) rigid PU with different SiC contents.

The thermal property of pure PU and its composites with SiC were examined by the DSC technique. The DSC graphs of pure flexible as well as rigid-PU and its composites with different SiC contents are shown in Fig. 7(a) and (b). A small transition temperature at a lower temperature indicates the T_g, SS_ and in this work, it is observed at −60 °C for both flexible, and rigid-PU matrices. Increasing SiC loading has no significant effect on T_g, SS_ of both flexible, and rigid-PU composite samples as provided in Table 3. This can be explained as the SiC-microparticles do not influence polypropylene glycol (polyol) segment motion in PU structure. The incorporation of particles can disrupt the entangled network of polymer chains, resulting in a breakdown and hence a slight decrease in glass transition temperature for both types of samples [[47], [48], [49]]. However, T_g, SS_ of rigid-PU, and SiC composites are not observed when SiC content is loaded from 3 to 14 v/v%. This may be attributed to SiC content does not influence the cross-linking and chain decomposing of rigid PU. For rigid-PU samples, broader endotherms peak at high SiC content could be indicated as a little degree of order within domains of the hard segment. At lower SiC content, endotherm peaks are narrower and highly intense than the higher content, indicating a high hard segment-crosslinking. It could be suggested that the high amount of SiC obstructed the molecular chain movement in the cross-linking structure in rigid PU composites. For the hard segment, the T_g, HS_ of both flexible as well as rigid-PU samples are provided in Table 3. T_g, HS_ present around 44 °C for flexible-PU composites. While T_g,HS_ slightly decreases from 44 °C to 39 °C when SiC content increased in case of rigid PU composites. The melting enthalpy is likely enhanced with increasing SiC content for flexible-PU composites, revealing the crystalline phase increased with increasing SiC content and improved the hard-segment phase separation in a flexible-PU structure. The melting enthalpy, in contrast, begins to reduce as SiC content rises for rigid PU samples which can be related to the peak of the broad endotherm. Adding higher SiC decreased the melting enthalpy because of the incorporation of micro fillers, catalyzing or enhances the polymerization of rigid PU matrix. This is result of the ceramic's viscosity-increasing effect on thermosetting materials, which is attributed to an enhancement of the curing temperature [48,50]. Furthermore, endothermic temperature (T_endo_) has no trend when SiC content was increased while it was decreased with loading SiC fillers as revealed in Table 3.

**Table 3:** Thermal properties of flexible as well as rigid-PU elastomers with different SiC contents.

Flexible Polyurethane				Rigid Polyurethane			
SiC content (v/v%)	T_g,SS_ (°C)	T_g,HS_ (°C)	T_endo_ (°C)	SiC content (v/v%)	T_g,SS_ (°C)	T_g,HS_ (°C)	T_endo_ (°C)
0	−60	45	68	0	−60	44	67
3.79	−60	45	67	3.97	N/A	42	60
8.13	−61	43	70	8.51	N/A	42	58
13.18	−61	44	66	13.75	N/A	39	60

## 4 Conclusion

Flexible as well as rigid-PU-/SiC composites successfully fabricated using solution casting process. Two phases were confirmed from SEM images of processed samples i.e., the matrix and SiC-microparticles. Adding SiC-filler into both flexible as well as rigid -PU matrices can improve the TC of the composites. Thermal conductivity of flexible-PU and rigid-PU/SiC composites were enhanced by 106% and 87%, respectively. The developed model is based on the mechanical model closely fitted to the experimental TCs, predicting the TC values of PU composites. It is found that the increasing tendency in TC is limited by the high viscosity of the polymer matrix and hence the agglomeration of fillers at higher loading. Furthermore, adding SiC-microparticles from 3 to 14 v/v% did not significantly influence the other thermal properties of both flexible, and rigid-PU composites. Various applications may be accessible, such as electrical and electronic devices and their packaging, heat management, etc., with good thermal properties’ composites through this method.

## Author contribution statement

Patcharapon Somdee, Manauwar Ali Ansari: Conceived and designed the experiments; Performed the experiments; Analyzed and interpreted the data; Contributed reagents, materials, analysis tools or data; Wrote the paper.

Tamas Szabo: Conceived and designed the experiments; Contributed reagents, materials, analysis tools or data.

Kalman Marossy: Conceived and designed the experiments; Performed the experiments; Contributed reagents, materials, analysis tools or data.

## Data availability statement

Data will be made available on request.

## Declaration of competing interest

The author(s) declare no competing interests.
